# An electronic health record based model predicts statin adherence, LDL cholesterol, and cardiovascular disease in the United States Military Health System

**DOI:** 10.1371/journal.pone.0187809

**Published:** 2017-11-20

**Authors:** Joseph E. Lucas, Taylor C. Bazemore, Celan Alo, Patrick B. Monahan, Deepak Voora

**Affiliations:** 1 Social Science Research Institute, Duke University, Durham, NC, United States of America; 2 Department of Medicine, Duke University, Durham, NC, United States of America; 3 United States Air Force, Air Force Medical Operations Agency, Joint Base Lackland-Kelly, Texas, United States of America; 4 Center for Applied Genomics & Precision Medicine, Duke University, Durham, NC, United States of America; University of Miami School of Medicine, UNITED STATES

## Abstract

HMG-CoA reductase inhibitors (or “statins”) are important and commonly used medications to lower cholesterol and prevent cardiovascular disease. Nearly half of patients stop taking statin medications one year after they are prescribed leading to higher cholesterol, increased cardiovascular risk, and costs due to excess hospitalizations. Identifying which patients are at highest risk for not adhering to long-term statin therapy is an important step towards individualizing interventions to improve adherence. Electronic health records (EHR) are an increasingly common source of data that are challenging to analyze but have potential for generating more accurate predictions of disease risk. The aim of this study was to build an EHR based model for statin adherence and link this model to biologic and clinical outcomes in patients receiving statin therapy. We gathered EHR data from the Military Health System which maintains administrative data for active duty, retirees, and dependents of the United States armed forces military that receive health care benefits. Data were gathered from patients prescribed their first statin prescription in 2005 and 2006. Baseline billing, laboratory, and pharmacy claims data were collected from the two years leading up to the first statin prescription and summarized using non-negative matrix factorization. Follow up statin prescription refill data was used to define the adherence outcome (> 80 percent days covered). The subsequent factors to emerge from this model were then used to build cross-validated, predictive models of 1) overall disease risk using coalescent regression and 2) statin adherence (using random forest regression). The predicted statin adherence for each patient was subsequently used to correlate with cholesterol lowering and hospitalizations for cardiovascular disease during the 5 year follow up period using Cox regression. The analytical dataset included 138 731 individuals and 1840 potential baseline predictors that were reduced to 30 independent EHR “factors”. A random forest predictive model taking patient, statin prescription, predicted disease risk, and the EHR factors as potential inputs produced a cross-validated c-statistic of 0.736 for classifying statin non-adherence. The addition of the first refill to the model increased the c-statistic to 0.81. The predicted statin adherence was independently associated with greater cholesterol lowering (correlation = 0.14, p < 1e-20) and lower hospitalization for myocardial infarction, coronary artery disease, and stroke (hazard ratio = 0.84, p = 1.87E-06). Electronic health records data can be used to build a predictive model of statin adherence that also correlates with statins’ cardiovascular benefits.

## Introduction

Atherosclerotic cardiovascular disease (ASCVD) is a leading cause of morbidity and mortality[1] caused, in part, by elevated low-density lipoprotein cholesterol (LDLc).[2] Lowering LDLc with statins is a cornerstone of ASCVD risk reduction.[2] The powerful effects of statins have resulted in a sustained reduction in the prevalence and mortality of ASCVD over the past three decades due to better control of cholesterol levels.[3] Based on current guidelines[2], approximately 56 million US adults are eligible for statin therapy.[4] However, up to 50% of patients prescribed statin therapy are non-adherent after one year,[5–7] that is associated with higher LDL-c, costs[8], and cardiovascular mortality, hospitalizations, and revascularization procedures [6].

Medication non-adherence is a complex problem caused by patient, provider, and health system-based obstacles and can be divided into primary (i.e. when a patient fails to pick up a prescription) and secondary non-adherence (i.e., when a patient does not refill an initially filled medication).[9] While several prior studies have determined variables that contribute to non-adherence, many of the studies do not evaluate the ability of these variables to predict statin adherence[10]. Therefore, improved models to predict medication non-adherence are needed to develop more effective patient-directed interventions[11]. Prior attempts to understand the factors that contribute to statin adherence have been limited due to these studies’ use of a restricted panel of input variables[10] that limits their predictive performance. Studies using larger sets of initially available patient, pharmacy, and physician characteristics also have had limited predictive capabilities (c-statistics < 0.65) for primary[12] or secondary medication non-adherence[13, 14]. As consequence of the poor performance of some electronic health records (EHR) based models, some have suggested that it may easier to look at past medication taking behavior[15] or early statin refill behavior[13] to predict long-term statin adherence. Although waiting for patients to stop taking their medications before delivering an intervention is a pragmatic approach, a predictive (or pre-emptive) approach employed at the time of statin prescription may be more effective in ensuring medications adherence. Finally, none of the current approaches to predict statin adherence have attempted to provide any clinical correlates of their predictions with respect to cardiovascular disease outcomes.

The availability of EHR data as opened new opportunities to model a variety of health outcomes. Typically, models utilizing this data source generally include patient records from administrative databases that make use of *a priori* patient information including ICD codes, pharmacy data, or other described characteristics[16]. The concept of medical care to characterize patient disease and outcomes is not new, and several models have been developed and validated based to predict patient outcomes.[16, 17] The transition to EHR data greatly expands the number of potential predictors for a particular health outcome by capturing a potentially more granular picture of patients. As an example, the use of EHR data has identified distinct subsets of heterogeneous disorders such as Type II diabetes[18]. However, the abundance of readily available, often incomplete, and non-standardized electronic medical data adds “noise” that complicates efforts to develop parsimonious models with high predictive power.[13] [19] As with genomics, for which the ability to capture increasingly larger orders of magnitude of data spurred the development of novel computational and statistical tools to analyze that data, the field of EHR analytics is rapidly evolving.[20, 21] Very few studies using the EHR to develop predictive models have focused on medication adherence.[22] Therefore, in this study we used EHR data from a well-defined cohort of patients cared for by the Military Health System (MHS) to build a predictive model of statin adherence in patients who have initiated statin therapy. We aimed to 1) develop an informative model for statin nonadherence and 2) assess the relationship between predicted statin adherence and changes in LDLc and hospitalizations related to myocardial infarction, coronary artery disease, and stroke during follow up. Our overall hypothesis was that patient characteristics embedded in the EHR could be used to identify patients with statin non-adherence and poorer cardiovascular prognosis.

## Methods

### Overview

The overall goal of this study was to use readily available data within an EHR to build a predictive model for adherence to statin medications in patients receiving their first statin prescription between 2005–2006. We first created a cohort of patients who filled their first statin prescription. We used data from the United States (US) Military Health System (MHS), which maintains billing, laboratory, and pharmacy claims data for TRICARE beneficiaries (active duty, retirees, and dependents of the US armed forces military) gathered from military treatment facilities across the US. Data from the two years leading up to a first statin prescription were utilized to develop a predictive model of 1) hospitalizations for cardiovascular disease and 2) statin adherence over the subsequent five years. The statin adherence model was then used to test for associations with changes in cholesterol levels and cardiovascular disease hospitalizations over the same time period (S1 Fig). The data used in this analysis was de-identified by the US Air Force and securely transferred to Duke University servers for subsequent analysis under a Data Use Agreement. The Duke University School of Medicine Institutional Review Board (protocol # Pro00046360) determined that these analyses were exempt research and did not meet the definition of human subjects research. An anonymized, analytical dataset (after data cleaning) is available in the Supplemental Information (S1 File).

### Data sources

Data for this study was collected from the MHS Management Analysis and Reporting Tool (M2)[23], which contains patient Military Treatment Facility (MTF) demographic data as well as inpatient and outpatient health care service elements provided by the MTFs (direct care) and civilian providers, hospitals, and managed care support contractors outside of MTFs (purchased care). The Pharmacy Data Transaction Service (PDTS) as well as the MHS Composite Health Care System provided pharmaceutical and laboratory data, respectively. All dates were truncated at the level of the month to help maintain patient privacy.

### Inclusion criteria

We defined the study cohort to be statin-naïve (defined as no prior statin refill in the prior 12 months) MHS adults between 18 and 65 years of age and continuously enrolled in TRICARE PRIME for at least 5 years beginning 01 January 2005. Eligible patients were prescribed their first (defined as PDTS ‘New Refill Code’ = 00, indicating first prescription fill) statin medication between 01 January 2005 and 31 December 2006. National Drug Codes (NDC), were used to define statins (S1 Table). Patients were counted only once and were included in the cohort irrespective of medication refills, discontinuation, or switches in statin drug class during the subsequent follow up period.

### Exclusion criteria

The initial dataset provided by MHS included 449 895 unique patients; however ~140K of these were treatment naïve on the date of statin initiation identified by MHS. Because of the difference in data collection before and after that date (see below), we limited our analysis to only these 140K. In addition, patients who filled a statin prescription in the preceding six months, received a prescription for a duration other than 30, 60, 90, or 180 days, or had less than one year of follow up were excluded from the analysis. In total, these filters removed less than 1 percent of the remaining patient population.

### Baseline data prior to first statin fill

Demographic, coding, pharmacy, and laboratory data from up to 2 years prior to the first statin fill were gathered from each patient. (Table 1)

**Table 1:** Electronic health record (EHR) data collected from each patient prior to 1^st^ statin fill.

Data type	Data collection period relative to 1^st^ statin filll	Description	Comments
Demographic	n/a	Sex, age, ethnicity, race, marital status, and pharmacy coverage program smoking	
Coding	1 year	Health Care Common Procedure Coding System (HCPCS) and International Classification of Disease (ICD) codes for inpatient admitting diagnosis, up to 10 additional diagnoses, and up to 20 procedures codes per encounter	Gathered from inpatient and outpatient encounters from direct and purchased care.
Laboratory	2 years	LDL cholesterol, creatinine, creatinine kinase, HDL cholesterol, total cholesterol and triglycerides	Most recent prior to initial fill defined as baseline
Prescription	1 year	NDC code, quantity filled, product strength, and days’ supply	Gathered for statins and concomitant medications

The longitudinal and repeated nature of EHR data presents challenges and opportunities for predictive modeling.[22] In order to characterize the extent to which a particular code is relevant for a given individual for each of the data types (pharmacy, laboratory, and coding),we counted the number of times that code appears in the medical record prior to statin initiation. Therefore instead of simply looking for the presence/absence of a particular drug (e.g. atenolol), laboratory value (e.g. LDL cholesterol), or diagnostic code (e.g. diabetes) we instead count the number of times a drug, code, or laboratory measure appears in the patients medical record in the time frame leading up to the 1^st^ statin fill. Patients with a higher number of appearances of a data type in the medical record may provide additional granularity about that patient above simple presence/absence. We computed count matrices for each data type (laboratory, prescription, and coding data) for the six months up to (and including) the first statin fill. This process generated 26 291 potential independent variables (i.e. counts) for use in predicting future statin adherence behavior.

For all laboratory data we used for each patient the most recent value measured before or at the time of first statin fill. For all pharmacy data, in order to generate an estimate of a patient’s prior adherence to non-statin medications, we additionally generated a prior percent days covered (PDC, see *Outcome definitions* for further description of PDC) for each non-statin fill date. Prior PDC was computed for each medication and each patient using the number of days from the first prescription of the medication in the dataset to the statin start date. Note that, because we are interested in adherence after the *first* prescription of a statin, there are no statins in the list of medications for which we have prior PDC. Prior PDC was only computed for a particular patient/medication pairing if there were at least 2 months of data to use for computation.

For all baseline data, we filtered out those variables that were observed in less than 100 patients.

### Follow up data after first statin fill

Follow up pharmacy data for all statin refills for each subject were recorded as a surrogate for non-statin adherence. Follow up laboratory data included total, LDL and HDL cholesterol and triglycerides. Follow up hospitalization data from both direct care and purchased care were collected for the following diagnoses: myocardial infarction, coronary artery disease (includes codes for unstable angina, ischemic heart disease, angina pectoris), kidney disease, and stroke (S2 Table).

### Outcome definitions

We defined statin adherence using the percent days covered (PDC), as this is increasingly becoming the preferred metric for medication adherence by Centers for Medicare and Medicaid. The PDC was calculated from the time of initial statin fill as the start date and the last recorded laboratory, pharmacy, or coding data element in the dataset because 1) statins are prescribed as long-term medications and 2) this model should account for patients who stop receiving care through the MHS during the follow up period as they will no longer appear in the dataset. Based on these dates, PDC was computed as the ratio of total days of filled statin medication (of any type) to the number of days of follow-up. From this data, binary indicators identifying (i) patients who filled their first refill (or not) and (ii) patients with PDC > or ≤ 80% were computed. For models that incorporated an indicator of whether the first statin refill was filled we also calculated a follow-up PDC beginning at the end of the course of the first prescription fill and not counting the pills in the first prescription.

To assess the proximal, pharmacologic effects of statin therapy, we focused on cholesterol data. Binary indicators of decreased or non-decreased cholesterol values were computed by comparing the last value measured before or on the statin start date to the first value measured at least 2 months subsequent to the statin start date.

To assess the more distal, clinical effects of statin therapy we examined hospitalization data for the presence of diagnoses that have been shown to be reduced by statin therapy, including acute myocardial infarction, stroke and coronary artery disease, as well as a composite of these diagnoses within 1 year of the statin start date.

### Statistical analysis

#### Overview

Based on our study design, we determined the *threshold date* to be the date on which a statin prescription was first filled (S1 Fig). Time 0 was set to this threshold date for each patient independently. The character of the data (particularly which codes were collected) changes at this date; all predictive models and analyses of statistical association use data from before the threshold to predict occurrences after. Initial data exploration suggested some potential biases in this type of observational dataset for patients who are 1) more likely to interact with the health care system and 2) have a higher disease burden/cardiovascular risk and are therefore more likely to be adherent to their statins. To overcome these biases we used two complementary approaches. First, we included the total number of interactions with the health system–broken down by labs, drugs and medical codes–as covariates in all models. Second, in order to account for the risk of cardiovascular disease, we generated a disease risk model to account for differences in patient comorbidities at the threshold date. We generated this disease risk model by first applying a dimension reduction technique (see below) to generate independent sets (or “factors”) of correlated baseline variables and used these factors to predict the likelihood of poor outcomes (defined as a composite of myocardial infarction, stroke, kidney disease, coronary artery disease) in the follow up period. The output of this model (i.e. risk of subsequent disease), along with the baseline patient and statin characteristics, were used as covariates in the statin adherence model. Last, in order to investigate the potential clinical utility of a statin adherence model we used the predicted statin adherence to test for associations with 1) cholesterol outcomes and 2) cardiovascular disease outcomes–two outcomes relevant to statin therapy.

#### Reducing the complexity of EHR data

Because many aspects of EHR data co-occur on patients’ charts, there is a high degree of correlation in the data. As a consequence, there is a certain level of redundancy in EHR data where some data elements can be substituted by others that are correlated with it. Furthermore, by modeling the correlation between data elements within the EHR dataset we can dramatically reduce the number of variables being tested to generate predictive models. Correlation between variables can also be used to collapse correlated variables into meaningful groupings or “factors” that may allow a clinical interpretation of the combined, correlated data elements. In this analysis, we collapsed each of the variables in the EHR dataset into 30 individual Factors (see S2 Table for lists of codes represented by each Factor). Last, for each Factor (1–30), each patient in the dataset can be assigned a factor “score” that represents the aggregate weight of each of the data elements within that factor. Factor scores are quantitative trait that we subsequently used to predict 1) disease risk and 2) statin adherence in the sections below.

#### Modeling disease risk

Patient and statin characteristics as well as factors scores described were considered as potential predictors. We used coalescent regression[24] to build the disease risk model using a binary outcome of event (hospitalization for kidney disease, myocardial infarction, coronary artery disease, and stroke) within 1 year. (Experimentation using standard Cox proportional hazard models did not improve results.) Coalescent regression is intended to borrow strength across similar disease outcomes and provides results that are more robust when the outcomes are related–particularly in the case of rare events. We use 30-fold cross-validation to assess model accuracy. Classification results for an indicator of event occurrence within three years following statin initiation are shown in S2 Fig. Kaplan-Meyer survival curves for tertiles of risk based on the disease risk model are shown in S3 Fig.

#### Modeling statin adherence

We again used patient and stain characteristics and the 30 factors for predictive modeling for statin adherence. We first tested for association between each of these independent factors and statin adherence using regression to control for all patient/statin characteristics as well as for the overall disease risk variable–obtained as described in section “*Modeling disease risk”* above. We then used random forests[25] to build models to predict adherence to statin therapy (PDC > 80%). The advantages of random forest classifiers include the ability to accommodate nonlinear relationships, interactions between variables, and efficiency in handling multi-dimensional data. However, because random forests are highly susceptible to overfitting, we used 30-fold cross-validation to generate estimates of accuracy for out-of-sample prediction. To improve our model, we also considered a model that also included first statin prescription refill as a potential predictor. In order to assess whether our statin adherence model adds to our ability to predict adherence above and beyond first refill behavior, we regressed statin adherence on our previously developed risk score and on an indicator of whether a first refill was purchased. For this model, we recomputed PDC for a time window beginning after the first prescription had completed. Last, to understand the relative importance of each variable for predicting PDC, we calculated the cross-validated change in area under the ROC curve by withholding that variable from the analysis.

#### Cholesterol and cardiovascular hospitalization outcomes

To test the association between predicted statin adherence and cholesterol outcomes, we used Pearson correlation for the association between the statin adherence risk score and (i) change in LDL cholesterol and (ii) change in total cholesterol. To test the association between predicted statin adherence and hospitalizations for CVD, we used Cox proportional hazards regression while controlling for patient and statin characteristics as well as for the predicted disease risk.

## Results

After applying our inclusion, exclusion, and filtering criteria the resulting analytical dataset had 850 count (laboratory, pharmacy, coding) variables, 106 laboratory value variables, 874 prior PDC values, ten patient and statin prescription characteristics (Table 2) on 138 731 patients who filled their first statin prescription. In a univariate analysis, many of these baseline variables were associated with PDC. (Table 2 and S4 Fig) Interestingly, however, prior adherence to non-statin medications was not associated with adherence to statins. (S4 Fig) To further characterize this cohort, we applied a dimension reduction technique to identify 30 sets of correlated baseline characteristics (or “factors”). Interpretation of the underlying patient disease(s) that are represented by each factor is not straightforward. However, meaningful interpretations of the most heavily loaded variables within each factor are apparent for most factors (S3 Table).

**Table 2:** Baseline patient and statin characteristics of cohort (N = 138731).

	# missing	Statin adherent (14328)	Statin non-adherent (124403)	p-value	CVD event	CVD event-free	p-value
Age, years	0	54	49	<1e-20	54	51	<1e-20
Female	15	0.43 (6093)	0.43 (53135)	0.677	.41 (413)	.42 (44174)	.216
Black	0	0.08 (1141)	0.08 (9847)	0.84	.09 (93)	.08 (8099)	.111
Tobacco	0	0.05 (736)	0.04 (5418)	1.69E-05	.1 (102)	.05 (4766)	2.22e-16
Follow up (days)	0	1325	1260	<1e-20	1270	1399	<1e-20
Strength (mg)	8693	46	44	0.000923	66	48	1.44e-14
Days Supply	0	69	67	2.23E-17	64	66	.00143
Number of labs	0	3.8	3.5	0.00018	2.5	3.9	9.48e-9
Number of concomittant drugs	0	5.0	3.8	<1e-20	4.6	4.6	2.78e-5
Number of codes	0	1.7	0.9	<1e-20	.08	1.1	8.89e-6

In our cohort we defined statin adherence as PDC > 80% because this cutoff is associated with reduction in CVD risk.[26] Based on this cutoff, we found that a minority of patients (14 328, 10.3%) met this strict criterion. Compared to patients who were non-adherent to statin therapy, those that were adherent were younger, female, smokers, with longer duration follow up, and had more frequent interactions with the health care system. (Table 2) In addition, higher initial statin prescription dose and days’ supply were both higher in those who were adherent to statin therapy. (Table 2) Of the 30 baseline factors identified through dimension reduction in our cohort, several were significantly associated with statin adherence (Table 3). Interestingly, patients with diabetes (Factor 3) or established cardiovascular disease (Factors 1 and 8) had higher statin adherence, perhaps reflecting a secondary prevention population. Conditions associated with lower adherence may reflect statin’s known side effects on musculoskeletal symptoms such as chronic pain (Factors 2, 4, 7, and 12).

**Table 3:** Groups of electronic health record codes and their association with higher or lower statin adherence.

Groupings of characteristics that promote higher statin adherence			
**Electronic health record Grouping**	**Clinical Interpretation**	**Top characteristics**	**P-value**
Factor 1	Atrial fibrillation	AtenololAtrial fibrillation	**<0.001**
		Sciatica	
		Hypokalemia	
		Infective otitis externa	
		Hytrin	
		Palpitations	
		Ecotrin	
		Muscular strength training	
		Prochlorperazine	
Factor 3	Diabetes/eye disease	Aspirin	**<0.001**
		Monlet lancets	
		Vitalet	
		Fundus photography	
		Senile nuclear sclerosis	
		Glynase	
		Accu-Chek	
		Softclix	
		Screening eye conditions	
		Multivitamin	
Factor 8	Congestive heart failure	Congestive heart failure	**0.0001**
		Furosemide	
		Lanoxin	
		Potassium chloride	
		Atrial Fibrillation	
		K-Dur	
		Klor-Con M20	
		Spironolactone	
		Coronary atherosclerosis of unspecified type of vessel	
		Coreg	

Factor 9	Sinus disease and allergies	Flonase	**0.008**
		Allergic rhinitis	
		Chronic rhinitis	
		Deep sea	
		Chronic sinusitis	
		Claritin	
		Dysfunction Eustachian tube	
		Serevent discus	
		Allergy, unspecified	
		Allegra	

Factor 11	Lisinopril	Lisinopril	**0.01**
		Vitalet	
		Softclix	
		Somatic dysfunction, thoracic	
		Impotence, organic origin	
		Cetaphil	
		Patient education	
		Lateral epicodylitis	
		Theophylline anhydrous	
		Caltrate-600 Plus	

Factor 13	Women’s health maintenance	Pelvic/clinical breast screen exam	**0.04**
		Screening-malignant neoplasms cervix	
		Other specified counseling	
		Screen malignant neoplasm-vagina	
		Screening Papanicolaou smear	
		Routine gynecological exam	
		Acquired absence genital organ	
		Screen malignant neoplasm-breast	
		Fecal occult blood test	
		Vaginitis	
**Groupings of characteristics that promote lower statin adherence**			
Factor 2	Physical medicine and rehabilitation	Traction/Mechanical modality	**<0.001**
		Physical therapy re-evaluation	
		Hot or cold packs therapy	
		Manual therapy service	
		Group therapeutic procedures	
		Ultrasound	
		Therapeutic activities	
		Therapeutic exercise	
		Physical therapy evaluation	
		Electrical stimulation	
Factor 4	Treatment for insomnia, pain	Ambien	**<0.001**
		Insomnia	
		Duragesic	
		Morphine sulfate	
		Psychotherapy w/ E/M services	
		Oxycontin	
		Ambien CR	
		Klonopin	
		Seroquel	
		Lumbago	
Factor 5	Eye disease	Presbyopia	**<0.001**
		Astigmatism	
		Regular astigmastism	
		Spectacle services	
		Hypermetropia	
		Myopia	
		Refractive state	
		Ophthalmologic services	
		Spectacle Services (Including prosthesis for aphakia)	
		Contact lens evaluation	
Factor 6	Treatment for upper respiratory infection	Zithromax	**<0.001**
		Robitussin A-C	
		Guaituss DM	
		Aerochamber	
		Guaituss AC	
		Tessalon perle	
		Acute bronchitis	
		Inhalation treatment for acute airway obstruction	
		Twice-a-day	
		Mucinex	
Factor 7	Treatment for pain; antibiotics	Hydrocodone w/ acetaminophen	**<0.001**
		Oxycodone Hcl- acetaminophen	
		Carisoprodol	
		Duragesic	
		Promethazine	
		Tizanadine	
		Lovenox	
		Hyosyamine sulfate	
		Cephalexin	
		Levaquin	
Factor 10	Asthma/ Chronic obstructive pulmonary disease	Asthma	**0.002**
		Albuterol sulfate	
		Ipratropium bromide	
		Aerochamber	
		Nebulizer treatment	
		Chronic obstructive pulmonary dis	
		Theophylline anhydrous	
		Advair diskus	
		Albuterol	
		Spiriva	
Factor 12	Musculoskeletal pain	Mobic	**0.03**
		Lumbosacral neuritis	
		Lumbar disc displacement	
		Arthropathy	
		Vioxx	
		Joint pain-pelvis	
		Osteoarthrosis	
		Somatic dysfunction lumbar	
		Myalgia and myositis	
		Sciatica	

**Fig 1:**
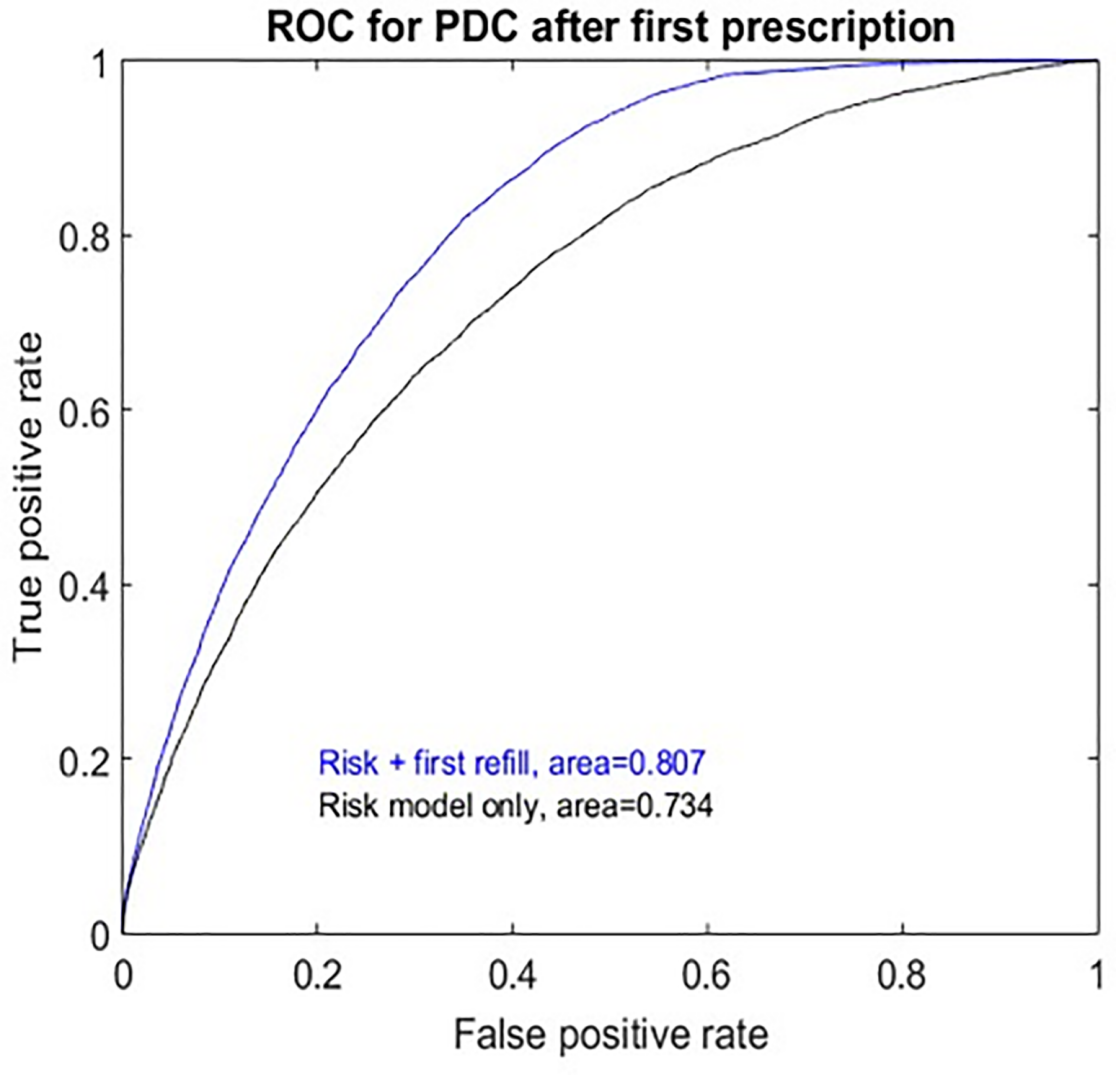
Performance of statin adherence models. The Receiver operating characteristics (ROC) curves for two models that predict statin adherence defined as percent days covered (PDC) greater than 0.8 during the follow-up period. The results of the risk only model uses random forest modeling and considers baseline demographics, statin prescription characteristics, disease risk predictions, and the ‘factors” resulting from dimension reduction to predict statin adherence. The “risk + first refill” model uses the same predictors as the risk only model but also considers whether or not the first statin prescription was filled and predicts statin adherence for the remaining time period after the first fill. The area represents the area under the ROC curve.

Because our overall goal was to develop a predictive model of statin adherence, we used random forest modeling to build a classifier composed of baseline patient/statin characteristics (Table 2) and the sets of correlated baseline variables (S3 Table) followed by 30-fold cross-validation. The c-statistic for this predictive model was 0.736 (Fig 1). Because first statin refill is a known predictor of subsequent statin refills in prior studies as well as in our own data (S4 Fig), we added first statin refill to our model after recalculating PDC for the time period following first statin refill. The resulting adherence risk model had a c-statistic of 0.81(Fig 1). To evaluate the relative importance of the baseline variables and factors in this model, we calculated the loss in predictive accuracy by withholding a particular variable and calculating the change in the area under the ROC curve (AUC, S5 Fig). The most important predictors were primarily related to duration of follow up, age, number of interactions with the health care system, and statin prescription characteristics. The top factors that contributed to the model were Factor 14: “gastrointestinal disease”, Factor 29: “Treatment of Hypertension”, Factor 28: “Hypertension/heart disease”, Factor 25: “Women’s health/menopause”

**Fig 2:**
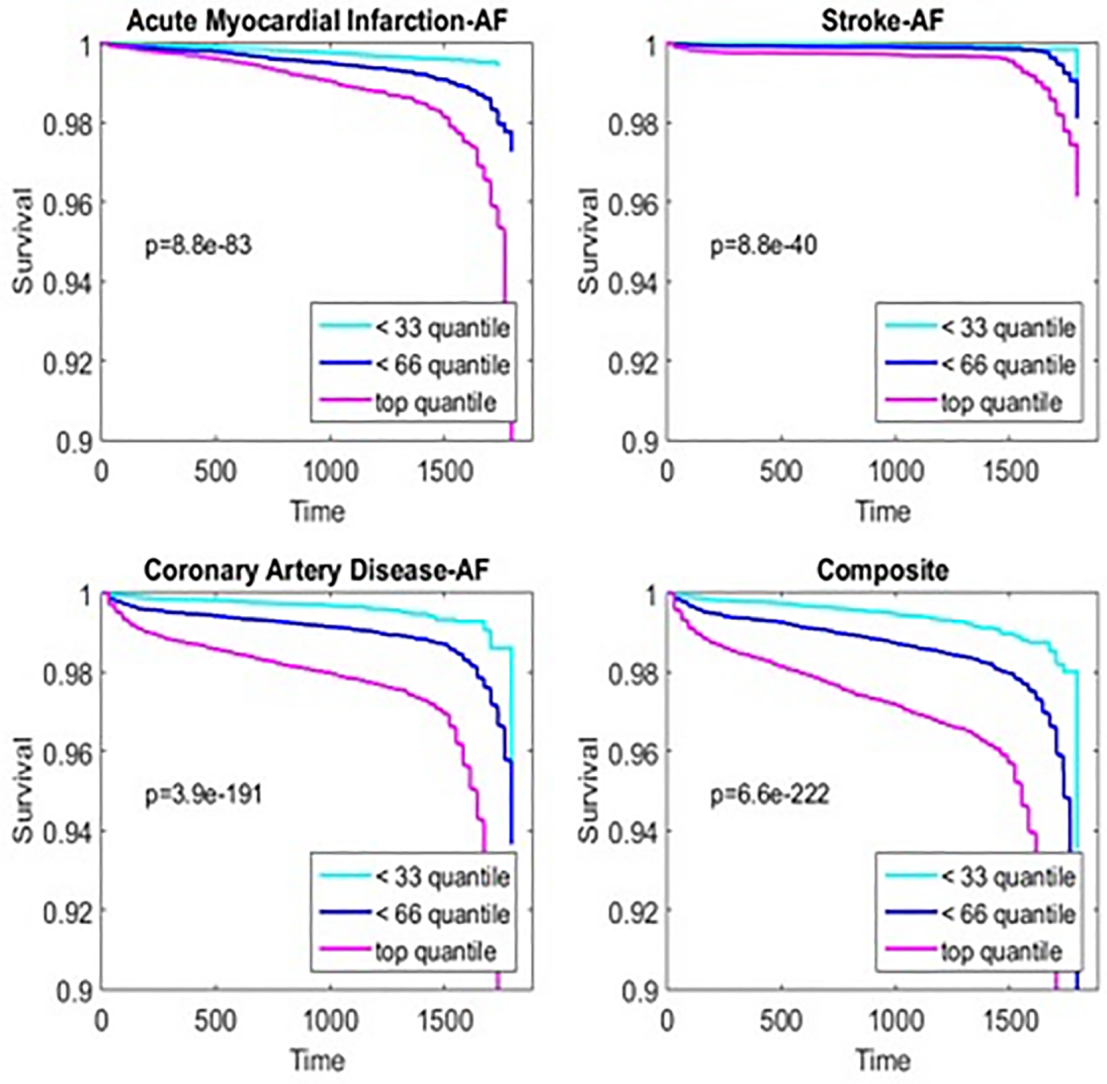
Predicted statin adherence and risk of cardiovascular outcomes. Predicted statin adherence was divided into tertiles of predicted statin adherence. The cumulative event free survival for each tertile of risk from Cox survival model is plotted for hospitalizations for acute myocardial infarction, stroke, coronary artery disease, or a composite of all three. P-values represent results of log-rank testing.

Statins are well-known to lower cholesterol and prevent CVD; therefore, to test the hypothesis that predicted statin adherence would also be informative on statin-responsive outcomes, we examined follow up data on LDL/total cholesterol and hospitalizations related to myocardial infarction, stroke, and coronary artery disease in the years following the initial statin prescription fill. We found that a higher predicted statin adherence was associated with a greater reduction in LDL and total cholesterol (correlations 0.14 and 0.15 respectively, p-values are < 1e-20) In addition, after considering baseline characteristics and disease risk, the statin adherence model was independently associated with an increased risk of hospitalizations for CVD related diagnoses. (Table 4 and Fig 2). Therefore, the predictions made by the statin adherence model are biologically and clinically relevant.

**Table 4:** Independent association between statin adherence model and cardiovascular disease hospitalization.

	Predicted Disease Risk		Predicted Statin Adherence	
	Hazard Ratio	p-value	Hazard Ratio	p-value
Acute Myocardial Infarction	1.22	< 0.001	0.81	< 0.001
Stroke	1.25	0.049	0.77	0.02
Coronary Artery Disease	1.49	< 0.001	0.81	< 0.001
Composite	1.34	<0.001	0.85	< 0.001

## Discussion

Statins are important medications for the reduction of cholesterol, incident and recurrent CVD, and CVD mortality. [1] However, the effectiveness of statins in preventing CVD in populations is eroded by non-adherence, with up to 50% of patients no longer taking statins one year after prescription.[5–7] Because interventions to improve medication adherence are time and resource intensive, predictive models to identify patients who will be non-adherent may allow for better allocation of limited resources. Prior attempts at predicting statin non-adherence have had limited success and have not examined clinical outcomes prevented by statin therapy. Using readily available administrative, pharmacy, and laboratory data from a single health system, our aim was to develop a predictive model for statin non-adherence and to assess the clinical impact of our predictions by examining cholesterol and CVD hospitalization outcomes. We found that we were able to develop a model that not only identified those patients at highest risk of statin-nonadherence but also patients with higher cholesterol and higher risk for CVD related hospitalizations during follow up.

Our approach to modeling statin non-adherence has several strengths. First, we used a dimension reduction approach that clustered the pharmacy, laboratory, and billing data into sets of correlated factors. This step facilitates implementation of our predictive model by allowing for missing data, which is common in EHR. Therefore, when attempting to predict the adherence of a new patient receiving a statin prescription, several data points can substitute for each other without a loss in model performance. Second, we link the results of our predictions to clinical outcomes such as decrease in LDL levels and hospitalizations related to CVD, both of which are both influenced by statin therapy. Thus, our model provides clinically relevant predictions. Third, by identifying specific patient clusters at higher (or lower) risk for adherence, our model lays the groundwork for future, personalized research to investigate the reasons for non-adherence. Patients who score high on non-adherence factors can be studied further to identify their reasons for non-adherence, and this information can be used to develop novel and tailored interventions to help patients to overcome these specific factors. Such a tool may be more effective than a “one size fits all” approach. One potential application for utilization of our model would be to use the readily available and highly predictive features such as first statin refill to identify and characterize the “at risk” patient and to use the clusters of HCPCS codes to tailor a specific intervention. The patient’s predicted risks can be reported on a continuous scale; therefore, a health care system can then compare a given patient to others and pick a threshold for intervention based on their resources (e.g., top 5, 10 or 20%).

Although identifying novel mechanisms for statin nonadherence was beyond the scope of this study, specific adherence clusters suggest novel areas that may underlie adherence. For example, patients characterized by chronic musculoskeletal pain (Factors 2, 12, 17, and 18) had a higher risk for statin non-adherence. Because statins are known to produce a range of musculoskeletal symptoms[27] it may be that patients with baseline, chronic pain are more susceptible to the effects of statins. Pre-emptive counseling, physical therapy, slow titration, or use of statins that are better tolerated may all be effective in mitigating this potential adverse effect. We acknowledge, however, that general variables are largely non-specific and by themselves do not necessarily allow for identification of the patients who would most benefit from targeted interventions to improve statin compliance. We anticipate that future refinement of these patient subgroups will ultimately lead to more precise estimates for statin non-adherence.

Despite the strengths of our approach, there were several limitations to consider. First, prescription refills are only a surrogate for medication adherence since we cannot know to what extent patients are taking their medications. The frequent use of “auto-refills” and 90-day supplies further makes true adherence difficult to assess. However, the correlations with LDL cholesterol and hospitalizations for CVD indicate that our predicted statin nonadherence was accurate in terms of their direction. Second, because we only focus on statin refills and hospitalizations for CVD we do not know to what extent our predictions are specific to statin therapy vs. general predictors of medication nonadherence. Third, we did not externally validate our model. However, our predictions are based on 30-fold internal cross validation. Therefore while we are confident with our internal validity, the external validity and generalizability of our model was not assessed. Last, our analysis focuses on statin adherence based on prescription fill data. An important group of patients who are non-adherent with statin therapy are those that do not fill their 1^st^ statin prescription (i.e. primary nonadherence). A definition of statin nonadherence that incorporates both primary and secondary nonadherence is critical for improving LDLc in a population.[28] Because we did not have access to prescriptions in our data we cannot evaluate the utility of our model in primary statin nonadherence in our data.

## Supporting information

S1 Fig Schematic of analytical approach. For each patient in the Military Health System the date of the first statin prescription fill was defined as T0. Panel A, Prior to T0, demographic, prior prescription (Rx), diagnostic codes from inpatient/outpatient encounters, and selected laboratory data were collected. These data were summarized using a dimension reduction approach (non-negative matrix factorization, see Methods) and used to build a predictive model of hospitalizations for coronary artery disease, myocardial infarction, stroke, or kidney disease during the follow up period after the first statin fill. Panel B, the same baseline data and dimension reduction approach along with the disease risk predictions from Panel A were used in a random forest prediction model to predict those patients who were more likely to be adherent with their statin prescriptions in the follow up period. Panel C, predicted disease risk and predicted statin adherence were tested for association with hospitalizations for adverse cardiovascular events and for cholesterol lowering.
(TIFF)

S2 Fig Performance of disease risk model for predicting hospitalizations. Receiver operating characteristics (ROC) curves showing predictive accuracy for the factor-regression model designed to predict disease occurrence within 3 years.
(TIFF)

S3 Fig Kaplan-Meier curves for each of disease risk tertiles. Using the model in S2 Fig, tertiles of predicted risk were identified and their cumulative event free survival plotted for each tertile. P-values represent log-rank test.
(TIFF)

S4 Fig Volcano plots of baseline predictors for statin adherence. Baseline disease codes (summarized into counts, see Methods), laboratory value data, and percent days covered (PDC) of non-statin medications prior to first statin fill were each tested for association with statin adherence. For each type of data (counts, laboratory, and prior PDC) the–log10 of the p-value is plotted on the y-axis and the direction and magnitude of effect plotted on the x-axis. Points represents the results of association testing for each potential predictor of interest. Points to the left of the vertical line in each plot are associated with higher statin adherence while those to the right are associated with lower statin adherence.
(TIFF)

S5 Fig First refill versus PDC. The distribution of statin adherence measured by the percent days covered (PDC) in the folloup period is plotted on the y-axis for two groups: 1) those that filled their first statin prescription (“Filled”) and 2) those that did not fill their first statin prescription (“Not filled”).
(TIFF)

S6 Fig Variable importance graph. The relative contribution of the top predictors used in the model is plotted. For the top variables in the model (y-axis) the change in c-statistic by withholding that variable is plotted on the x-axis.
(TIFF)

S1 Table List of statin medications. The list of statin medications and the national drug codes used to identify them is listed.
(XLSX)

S2 Table List of hospitalization codes. The international classification of disease diagnostic codes for hospitalizations used during follow up.
(XLSX)

S3 Table List of factors from dimension reduction analysis. The list of factors resulting from the dimension reduction analysis of coding, laboratory, and prescription refill data.
(XLSX)

S1 File Compressed/ZIP file archive. The following files/datasets were used in the analyses presented in this manuscript after data cleaning (see Methods).
File A contains the baseline variables listed in File B for all participantsFile C contains all of the laboratory variables listed in File D for all participantsFile F contains the medication possession ratio (as defined in Methods” for all medications listed in File GFile H contains the count matrix for each of the variables listed in File I for all participants.File J contains each of the outcome measures listed in File K for all participantsFile L contains the Factor Scores for each of the Factors listed in File M that were generated from the non-negative matrix factorization of the count matrix File H (see Methods).
(ZIP)
