# Supplementary figures and images for: An electronic health record based model predicts statin adherence, LDL cholesterol, and cardiovascular disease in the United States Military Health System
Source: PLoS One. 2017 Nov 20;12(11):e0187809. doi: 10.1371/journal.pone.0187809 (PMC5695792; doi:10.1371/journal.pone.0187809)

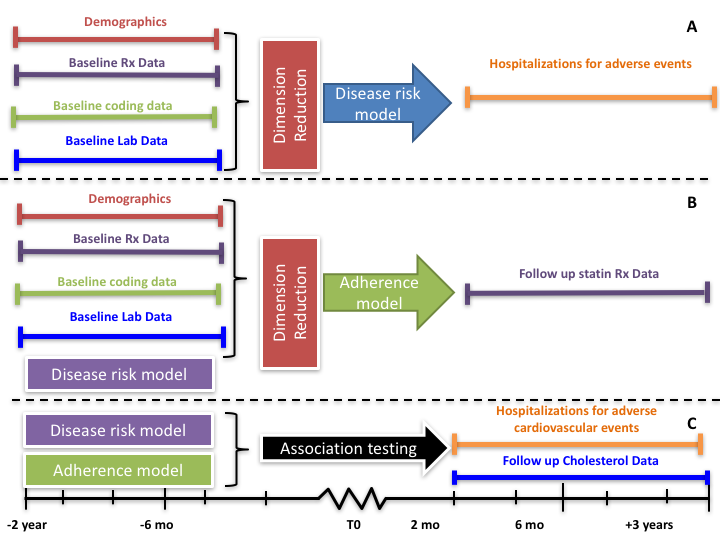

Supplement: S1 Fig — For each patient in the Military Health System the date of the first statin prescription fill was defined as T0. Panel A, Prior to T0, demographic, prior prescription (Rx), diagnostic codes from inpatient/outpatient encounters, and selected laboratory data were collected. These data were summarized using a dimension reduction approach (non-negative matrix factorization, see Methods) and used to build a predictive model of hospitalizations for coronary artery disease, myocardial infarction, stroke, or kidney disease during the follow up period after the first statin fill. Panel B, the same baseline data and dimension reduction approach along with the disease risk predictions from Panel A were used in a random forest prediction model to predict those patients who were more likely to be adherent with their statin prescriptions in the follow up period. Panel C, predicted disease risk and predicted statin adherence were tested for association with hospitalizations for adverse cardiovascular events and for cholesterol lowering. (TIFF) [file pone.0187809.s001.tiff]

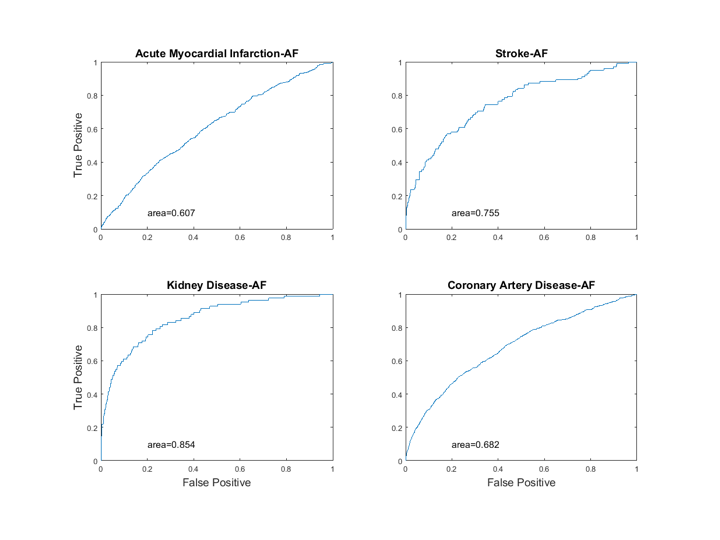

Supplement: S2 Fig — Receiver operating characteristics (ROC) curves showing predictive accuracy for the factor-regression model designed to predict disease occurrence within 3 years. (TIFF) [file pone.0187809.s002.tiff]

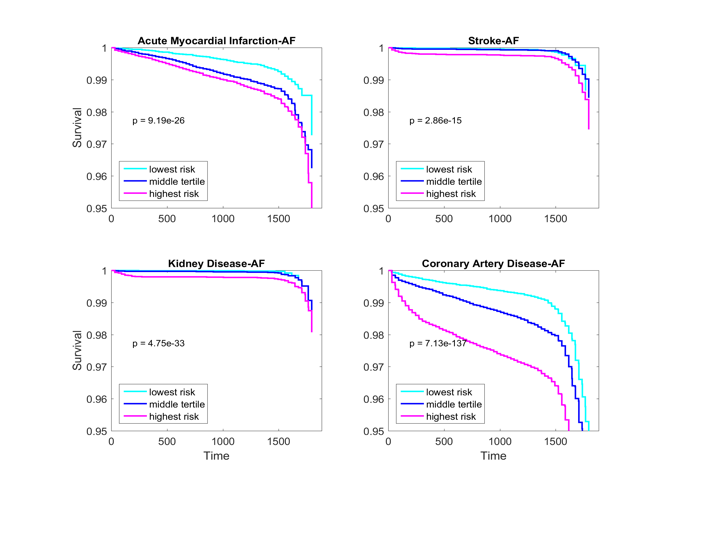

Supplement: S3 Fig — Using the model in S2 Fig, tertiles of predicted risk were identified and their cumulative event free survival plotted for each tertile. P-values represent log-rank test. (TIFF) [file pone.0187809.s003.tiff]

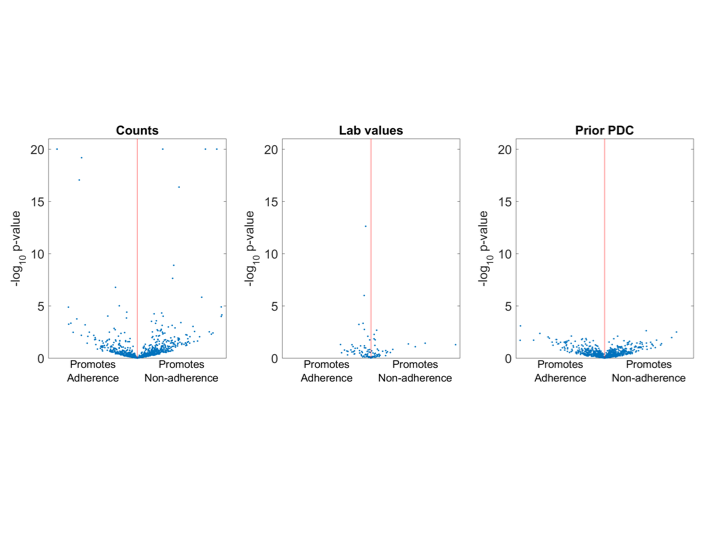

Supplement: S4 Fig — Baseline disease codes (summarized into counts, see Methods), laboratory value data, and percent days covered (PDC) of non-statin medications prior to first statin fill were each tested for association with statin adherence. For each type of data (counts, laboratory, and prior PDC) the–log10 of the p-value is plotted on the y-axis and the direction and magnitude of effect plotted on the x-axis. Points represents the results of association testing for each potential predictor of interest. Points to the left of the vertical line in each plot are associated with higher statin adherence while those to the right are associated with lower statin adherence. (TIFF) [file pone.0187809.s004.tiff]

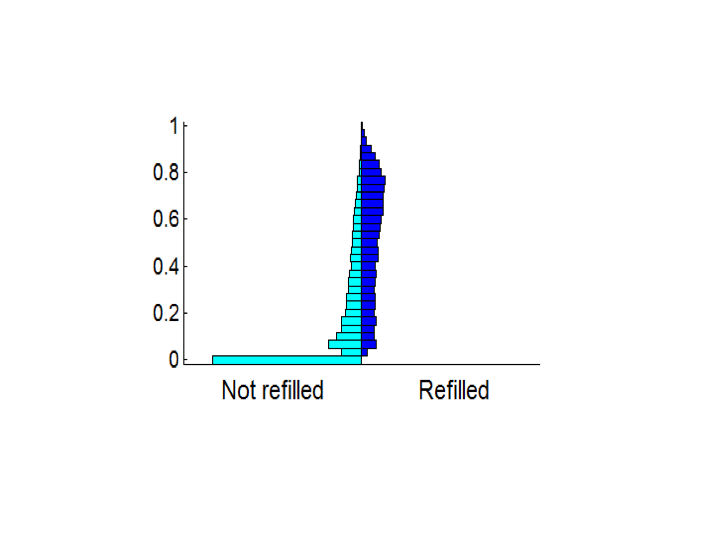

Supplement: S5 Fig — The distribution of statin adherence measured by the percent days covered (PDC) in the folloup period is plotted on the y-axis for two groups: 1) those that filled their first statin prescription (“Filled”) and 2) those that did not fill their first statin prescription (“Not filled”). (TIFF) [file pone.0187809.s005.tiff]

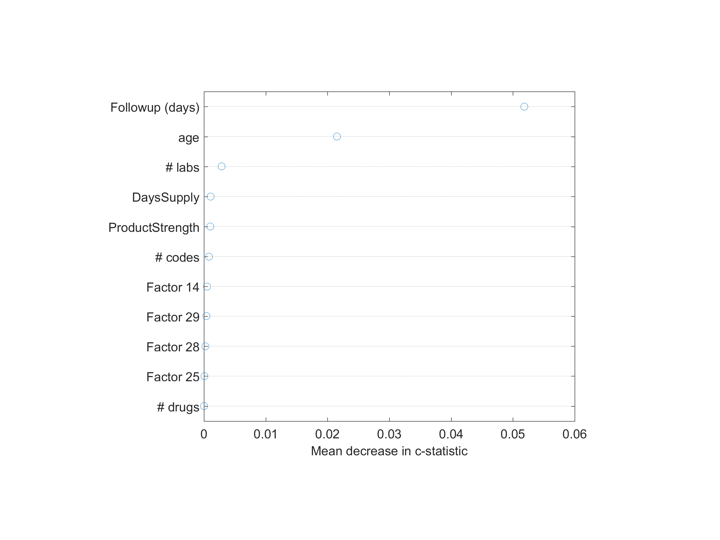

Supplement: S6 Fig — The relative contribution of the top predictors used in the model is plotted. For the top variables in the model (y-axis) the change in c-statistic by withholding that variable is plotted on the x-axis. (TIFF) [file pone.0187809.s006.tiff]
